# Functional analysis of 110 phosphorylation sites on the circadian clock protein FRQ identifies clusters determining period length and temperature compensation

**DOI:** 10.1093/g3journal/jkac334

**Published:** 2022-12-20

**Authors:** Bin Wang, Elizabeth-Lauren Stevenson, Jay C Dunlap

**Affiliations:** Department of Molecular and Systems Biology, Geisel School of Medicine at Dartmouth, Dartmouth College, Hanover, NH 03755, USA; Department of Molecular and Systems Biology, Geisel School of Medicine at Dartmouth, Dartmouth College, Hanover, NH 03755, USA; Department of Molecular and Systems Biology, Geisel School of Medicine at Dartmouth, Dartmouth College, Hanover, NH 03755, USA

**Keywords:** FRQ, phosphorylation, mutants, period length, temperature compensation, *Neurospora*

## Abstract

In the negative feedback loop driving the *Neurospora* circadian oscillator, the negative element, FREQUENCY (FRQ), inhibits its own expression by promoting phosphorylation of its heterodimeric transcriptional activators, White Collar-1 (WC-1) and WC-2. FRQ itself also undergoes extensive time-of-day-specific phosphorylation with over 100 phosphosites previously documented. Although disrupting individual or certain clusters of phosphorylation sites has been shown to alter circadian period lengths to some extent, it is still elusive how all the phosphorylations on FRQ control its activity. In this study, we systematically investigated the role in period determination of all 110 reported FRQ phosphorylation sites, using mutagenesis and luciferase reporter assays. Surprisingly, robust FRQ phosphorylation is still detected even when 84 phosphosites were eliminated altogether; further mutating another 26 phosphoresidues completely abolished FRQ phosphorylation. To identify phosphoresidue(s) on FRQ impacting circadian period length, a series of clustered *frq* phosphomutants covering all the 110 phosphosites were generated and examined for period changes. When phosphosites in the *N*-terminal and middle regions of FRQ were eliminated, longer periods were typically seen while removal of phosphorylation in the C-terminal tail resulted in extremely short periods, among the shortest reported. Interestingly, abolishing the 11 phosphosites in the C-terminal tail of FRQ not only results in an extremely short period, but also impacts temperature compensation (TC), yielding an overcompensated circadian oscillator. In addition, the few phosphosites in the middle of FRQ are also found to be crucial for TC. When different groups of FRQ phosphomutations were combined intramolecularly, expected additive effects were generally observed except for one novel case of intramolecular epistasis, where arrhythmicity resulting from one cluster of phosphorylation site mutants was restored by eliminating phosphorylation at another group of sites.

## Importance

Circadian rhythms, found in most eukaryotes, are based on cell-autonomous, auto-regulatory feedback loops in which negative elements feed back to depress their own expression by repressing the positive elements that drive their synthesis. In *Neurospora*, the WCC transcription activator drives the expression of FRQ, which complexes with FRH and CK1 to repress the DNA-binding activity of WCC by promoting phosphorylation at a group of residues of WCC. The phosphorylation status of FRQ determines the circadian period length, acting independently of effects of phosphorylation on FRQ half-life. Reflecting this dominant role of phosphorylation, FRQ is subject to substantial phosphorylation at over 100 sites in a time-of-day-specific manner. However, how this plethora of phosphoevents on FRQ controls its activity in a circadian cycle is still elusive, and prior work had shown limited effects of individual phosphosite point mutants. In this study, a series of *frq* mutants targeting multisite phosphorylation within domains of FRQ were generated and analyzed in order to define their roles in period determination. A clear pattern of period-altering effects was observed in these *frq* mutants; certain mutants display strong temperature compensation phenotypes, and interestingly, a novel epistatic relationship on rhythmicity between phosphogroups emerged.

## Introduction

Living organisms on earth are persistently under the influence of external light/dark cycles. To anticipate and, more importantly, better utilize these environmental cues, most organisms have evolved an internal cellular oscillator, the circadian clock, that integrates daily signals, such as light, temperature, and chemicals, to metabolism (Dunlap 1996; Dunlap and Loros 2006; Guo and Liu 2010; Wang *et al.* 2013; Larrondo and Canessa 2018; Diernfellner and Brunner 2020; Zhang *et al.* 2022). Circadian clocks regulate a wide variety of physiological and molecular events in eukaryotes and certain prokaryotes (Michael *et al.* 2015; Karki *et al.* 2020; Ding *et al.* 2021).

Unlike light and chemicals that only function as Zeitgebers to the core clock, the temperature can impact the core oscillator in several different ways: The period length of circadian clocks remains about the same across permissive temperatures—a phenomenon commonly called “temperature compensation” that allows the clock to make accurate time measurements while temperatures undergo large variations in nature; similar to light, both temperature pulses and steps can reset the oscillator, serving as an entrainment factor for the molecular clocks (Sweeney and Hastings 1960; Francis and Sargent 1979; Edery *et al.* 1994; Gooch *et al.* 1994; Liu *et al.* 1997); and finally, circadian clocks can only oscillate within a limited range of temperatures, outside which the clock will be frozen at a certain phase from which rhythmicity can be resumed if the organism is returned to permissive temperatures (Njus *et al.* 1977).

In *Neurospora*, *Drosophila*, and mammals, the core circadian oscillator comprises a transcription and translation-based negative feedback loop: Negative elements (FREQUENCY [FRQ], PERIODS [PERs], and CRYPTOCHROMES [CRYs]) bring about repression to their transcriptional activators, WC-1 and WC-2 in *Neurospora*, Clock (Clk) and Cycle (Cyc) in *Drosophila*, and BMAL1/Circadian Locomotor Output Cycles Kaput (CLOCK) in mammals, to terminate their own expression, thereby closing the circadian feedback loop (Guo and Liu 2010; Zhang *et al.* 2011). For example, in *Neurospora*, the White Collar Complex (WCC), a heterodimer comprised of WC-1 and WC-2, serves as the transcriptional activator for the pacemaker gene *frequency* (*frq*) by binding to one of the two DNA elements in the *frq* promoter: the *Clock box* (*C-box*) in the dark (Froehlich *et al.* 2003) or the *Proximal Light-Response Element* (*PLRE*) in the light (Froehlich *et al.* 2002). FRQ interacts with FRQ-Interacting RNA Helicase (FRH) and Casein kinase 1 to repress the transcription activity of WCC by promoting its phosphorylation at a group of residues (Aronson *et al.* 1994b; Lee *et al.* 2000; Cheng *et al.* 2005; Schafmeier *et al.* 2005; He *et al.* 2006; Hong *et al.* 2008; Guo *et al.* 2009; Shi *et al.* 2010; Guo *et al.* 2010; Cha *et al.* 2011; Hurley *et al.* 2013; Lauinger *et al.* 2014; Wang *et al.* 2019).

Protein phosphorylation, as the most common post-translational modification, has been implicated in regulating protein-DNA interaction, protein-protein interaction, protein turnover, enzymatic activity, and subcellular localization, all of which have been shown to control the operation of the circadian clocks (e.g. Luo *et al.* 1998; Diernfellner *et al.* 2009; Lipton *et al.* 2015; Robles *et al.* 2017; Narasimamurthy *et al.* 2018; Luciano *et al.* 2018). In the *Neurospora* clock, FRQ, the core pacemaker protein, undergoes dual molecular rhythms in total abundance and phosphorylation (Garceau *et al.* 1997; Liu *et al.* 2000; Ruoff *et al.* 2005). The dynamic phosphorylation status of FRQ is controlled by several kinases and phosphatases including the Casein kinases 1 and 2 (CK1 and CK2), Checkpoint Kinase 2 (PRD-4), Protein kinase A (PKA), Ca/CaM-dependent kinase (CAMK-1), and protein phosphatase (PP) 1, 2A, and 4 (PP1, PP2A, and PP4) (Yang *et al.* 2001, 2002; Brunner and Schafmeier 2006; Pregueiro *et al.* 2006; Dunlap 2006; He *et al.* 2006; Huang *et al.* 2007; Cha *et al.* 2008; Diernfellner *et al.* 2019; Wang *et al.* 2021). Newly expressed FRQ becomes progressively phosphorylated over time and is targeted eventually for degradation through the SCF-ubiquitin ligase-recruiting protein FWD-1 (He *et al.* 2003). FRQ undergoes extensive phosphorylation at over 100 residues in a time-of-day-specific manner (Garceau *et al.* 1997; Liu *et al.* 2000; Baker *et al.* 2009; Tang *et al.* 2009) that determines its activities, controls its binding partners, and, finally, leads to its inactivation (Baker *et al.* 2009; Larrondo *et al.* 2015). Quantitative mass spectrometry analysis reveals that phosphorylation of distinct regions of FRQ occurs at opposite phases of the clock, causing opposing effects on its activity and interacting partners over time (Baker *et al.* 2009; Tang *et al.* 2009). In vitro kinase assays revealed that CK1 and CK2 account for a large body of FRQ phosphorylation events (Tang *et al.* 2009). In addition to period determination at one temperature, FRQ phosphorylation and related kinases have also been implicated in temperature compensation of the clock across physiological temperature ranges (Aronson *et al.* 1994a; Pregueiro *et al.* 2005). For example, CK2 contributes to establishing the temperature compensation of the clock via FRQ phosphorylation at certain residues (Mehra *et al.* 2009). In a recent study, FRQ-CK1 interaction as well as CK1- and CK2-mediated FRQ phosphorylation has been noted for regulating the period length across temperatures (Hu *et al.* 2021). Temperature also controls the ratio of L-FRQ to S-FRQ, derived from different start codons used in translation initiation, which is crucial for maintaining rhythmicity at a low or high temperature (Liu *et al.* 1997; Diernfellner *et al.* 2005; Colot *et al.* 2005).

Mutagenetic analysis of all the plethora of phosphoresidues on FRQ becomes unavoidable and urgent in order to more fully understand their roles in controlling and fine-tuning the pace of the core oscillator. To this end, we engineered and investigated a large number of *frq* phosphomutants covering all the known 110 phosphosites; these were then progressively dissected into smaller clusters to discover the phosphogroups important for determining FRQ activity and thus period length. Taken together, the data show that eliminating certain phosphoclusters in the *N*-terminal and middle regions of FRQ mainly causes period lengthening while ablation of multisite phosphorylation at the C-terminus results in an extremely short period of 14–15 hours. Interestingly, impairing phosphorylation of a cluster of residues at the C-terminus of FRQ not only shortens the period but also leads to an overcompensated clock across a set of physiological temperatures; moreover, the elimination of certain phosphosites in the middle of FRQ leads to increased period lengths at elevated temperatures as well. Furthermore, unexpectedly, one group of phosphosites on FRQ can be epistatic to another in period determination.

## Materials and methods

### Growth conditions

All vegetative cultures were maintained on complete medium slants bearing 1 × Vogel's, 1.6% glycerol, 0.025% casein hydrolysate, 0.5% yeast extract, 0.5% malt extract, and 1.5% agar (Vogel 1956). Sexual crosses were performed on Westergaard's agar plates containing 1 × Westergaard's salts, 2% sucrose, 50 ng/ml biotin, and 1.5% agar (Westergaard and Mitchell 1947). Liquid culture medium (LCM) contains 1 × Vogel's, 0.5% arginine, 50 ng/ml biotin, and 2% glucose.

### *frq* mutant generation

To lower the cost of making a large number of *frq* mutants, a method described in Baker *et al.* (2009) was modified to use yeast homologous recombination-based integration of PCR fragments (Wang *et al.* 2014) bearing FRQ point mutations to restriction-digested *pCB05* in place of the QuickChange II Site-directed Mutagenesis Kit (Stratagene). Four primer sets were used as flanks to facilitate homologous recombination in a yeast strain (*FY834*) by which point mutations of *frq* were introduced from PCR primers. To introduce mutations to aa 1–214 of FRQ, two PCR reactions were performed: one with a forward primer “*frq* segment 1F” (5′-GAACCAGAACGTAGCAGTGTG-3′) and a reverse primer “#pA R” bearing a point mutation(s) to FRQ and the other using a forward primer “#pA F” which is reverse and complementary to “#pA R” and a reverse primer “*frq* segment 1R” (5′-GACGATGACGACGAATCGTG-3′), and then the two PCR products were co-transformed into yeast along with *pCB05* (Baker *et al.* 2009) digested with *Bst*XI and *Xho*I to create a circular construct. Similarly, to introduce mutations falling in aa 215–437 of FRQ, primers “*frq* segment 2F” (5′-GTGAGTTGGAGGCAACGCTC-3′) and “*frq* segment 2R” (5′-GTCCATATTCTCGGATGGTA-3′ were used for PCRs in combination with *pCB05* digested with *Xho*I to *Nru*I; “*frq* segment 3F” (5′-GTCGCACTGGTAACAACACCTC-3′) and “*frq* segment 3R” (5′-CAGCACATGT TCAACTTCAT CAC-3′) were designed for *pCB05* digested with *Nru*I and *Fse*I (FRQ aa 438–675), and “*frq* segment 4F” (5′-CACCGATCTTTCAGGAGACCCTG-3′) and “*frq* segment 4R” (5′-CACTCAGGTC TCAATGGTGA TG-3′) work for *pCB05* digested with *Fse*I *and Mlu*I (FRQ aa 676–989). If multiple phosphosites span two or more PCR segments mentioned above, corresponding restriction enzymes and primers encompassing the region were chosen and combined for recombination in yeast. All mutations were verified by cycle sequencing at the Dartmouth Core facility. The open reading frame of *frq* bearing 84 phosphomutations (*frq^84A^*) from (Baker *et al.* 2009) was custom-synthesized and purchased from Genscript, and to *frq^84A^*, additional 26 phosphosites identified in (Tang *et al.* 2009) were further mutated to Ala by PCR reactions using primer pairs bearing mutations to create *frq^110pA^*. All *frq* mutant constructs were targeted by homologous recombination to its native locus. Plasmids verified by cycling sequencing were linearized with *Ase*I and *Ssp*I and PCR-purified for *Neurospora* transformation. *Neurospora* transformation was performed as previously reported (Colot *et al.* 2006). The recipient strain used in transforming *frq* mutants is Δ*frq::hph*; Δ*mus-52::hph*; *ras-1^bd^*; *C-box luc at his-3*, and all *frq* mutants made in this study were in the *ras-1^bd^* genetic background (Belden *et al.* 2007) and bear a V5H6 tag at their C-termini and *frq-C-box*-driven codon-optimized firefly *luciferase* gene at the *his-3* locus (Gooch *et al.* 2008), except for the strains in Fig. 5, all of which bear *frq-C-box*-driven *luciferase* at the *csr-1* locus rather than *his-3*. These strains were constructed by crossing phosphomutants from Baker *et al.* (2009) to *frq-C-box-luc* at *csr-1*.

### Immunoprecipitation (IP)

IP was performed as previously described (Wang *et al.* 2016). Briefly, 2 mg of total protein was incubated with 20 μl of V5 agarose (Sigma-Aldrich, #7345) as indicated rotating at 4°C for 2 hours. The agarose beads were then washed twice with the protein extraction buffer (50 mM HEPES [pH 7.4], 137 mM NaCl, 10% glycerol, 0.4% NP-40) and eluted with 100 µl of 5 × SDS sample buffer heated at 99°C for 5 minutes.

### Lambda protein phosphatase-treatment of FRQ

V5H6-tagged FRQ was immunoprecipitated with 20 μl of V5 agarose (Sigma-Aldrich, Catalog #7345) from 2 mg of centrifugation-cleared lysate, FRQ-bound V5 agarose was thoroughly washed twice using the protein extraction buffer, and all supernatant was carefully removed by pipetting. To make a total reaction volume of 52 µl, 40 μl of H_2_O, 5 μl of 10×NEBuffer for Protein MetalloPhosphatases (PMP), 5 μl of 10 mM MnCl_2_, and 2 μl of lambda protein phosphatase (NEB, Catalog #P0753S) were added to the washed FRQ-coupled V5 resin. The mixture was incubated at 30°C for 30 minutes, and then 50 µl of 5 × SDS sample buffer was added and heated at 99°C for 5 minutes (Zhou *et al.* 2018).

### Western blot (WB)

For WB, equal amounts (15 μg) of cleared protein lysate were loaded per lane in an SDS-PAGE gel. FRQ, FRH, WC-1, and WC-2 antibodies were previously described (Garceau *et al.* 1997; Denault *et al.* 2001; Froehlich *et al.* 2002). Antibody against V5 (Thermo Pierce) was used at 1:5,000 dilution as the first antibody in WB (Wang *et al.* 2021).

### Phos-tag gel

To better resolve FRQ phosphorylation events, Phos-tag chemical purchased from ApexBio was added at the final concentration of 20 μM to the 6.5% SDS-PAGE Tris-Glycine gel with a ratio of 149:1 acrylamide/bisacrylamide (Wang *et al.* 2019).

### Luciferase assay

Luciferase assays were performed as previously described (Larrondo *et al.* 2012). 96-well plates with each well containing 0.8 ml of the luciferase assay medium were inoculated with conidial suspension and unless otherwise specified, strains in luciferase assays were cultured at 25°C and in constant light for 16–24 hours and then transferred to the dark at the same temperature for recording light signals. Bioluminescence signals were recorded with a CCD camera every hour, data were obtained with ImageJ and a custom macro, and period lengths of the strains were manually calculated. Raw data from three replicates are shown, and time (in hours) is on the x-axis while arbitrary units of the signal intensity are on the y-axis. In Fig. 4, the strains were synchronized at 20, 25, or 30°C plus light overnight and then transferred to darkness at the same temperature used in synchronization to monitor light production by a CCD camera. Strains in Fig. 5 were entrained at 25°C for two days on a 12/12 light/dark cycle before transferring to the dark at either 20, 25, or 30°C to monitor light production by a CCD camera. Luciferase assay medium contains 1 × Vogel's salts, 0.17% arginine, 1.5% bacto-agar, 50 ng/ml biotin, and 0.1% glucose. Except for Fig. 5 (see Fig. 5 legend for controls used), WT used in the luciferase assays was 661–4a (*ras-1^bd^*, *A*) that contains the *frq-C-box* fused to the codon-optimized firefly *luciferase* gene (transcriptional fusion) at the *his-3* locus.

## Results

### A mutagenetic strategy developed to progressively explore roles of the 110 phosphosites on FRQ

**Fig. 1.**
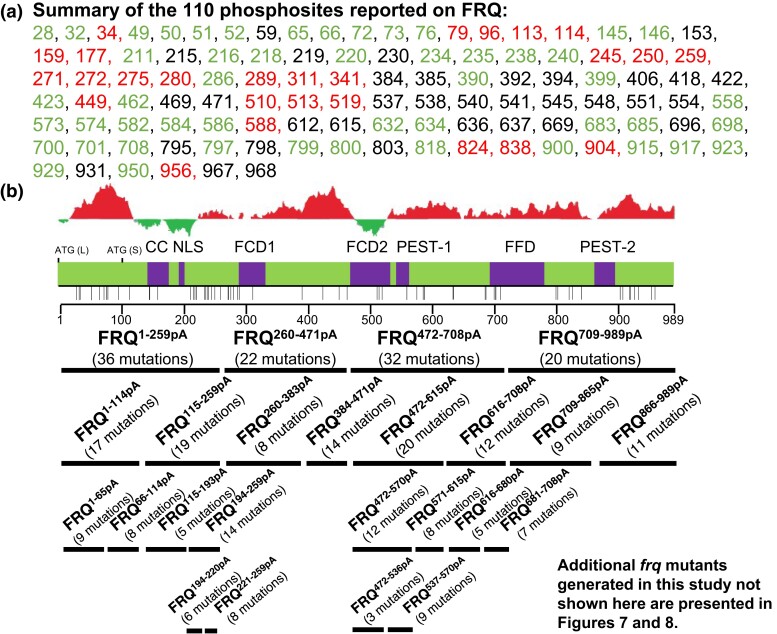
Summary of phosphosites reported on FRQ and *frq* phosphomutants generated in this study a) summary of the 110 phosphorylation sites from two publications (Baker *et al.* 2009; Tang *et al.* 2009). Numbers represent sites on FRQ at which phosphorylation occurs: Sites reported in (Baker *et al.* 2009), (Tang *et al.* 2009) and both (Baker *et al.* 2009) and (Tang *et al.* 2009) are in black, red, and green, respectively. b) *frq* phosphorylation mutants engineered and investigated in this study. Upper, schematic of FRQ. Each horizontal bar represents a *frq* mutant with phosphosites falling in the region of the bar mutated to Ala altogether while keeping phosphosites outside the region WT. The number of mutations introduced per mutant is in parentheses. ATG (L) is the first start codon used in translation resulting in the full-length FRQ; ATG (S) is an isoform of FRQ translated from the third translational start site (ATG) of the *frq orf*, 99 aa downstream of ATG (L); previously described domains on FRQ are in purple, including the following: CC, coiled-coiled domain; NLS, nuclear localization signal; FCD, FRQ-CK1 interacting domain; PEST-#, pest domains. FFD, FRQ-FRH interacting domain. Each vertical bar below FRQ represents a site phosphorylated by CK1, CK2, or CK1 and CK2 in vitro (Tang *et al.* 2009). Above the diagram is a structural complexity analysis of FRQ amino acids: Red peaks represent disordered regions while green is for structured domains.

A total of 110 phosphosites on FRQ (Fig. 1a) have been identified by mass spectrometry (Baker *et al.* 2009; Tang *et al.* 2009), but mutagenetic analyses have been conducted covering only some of these phosphosites. In this study, to screen phosphosites on FRQ impacting the pace of the circadian oscillator, we adopted a strategy successfully employed in a recent publication by which a small group of phosphoresidues from over 95 sites on WCC was identified for determining the repression of WCC and thereby the closure of the feedback loop (Wang *et al.* 2019). To this end, we engineered a series of *frq* mutants (replacing Ser/Thr with Ala) covering all the 110 phosphosites in a group manner (Fig. 1a and 1b) and then assayed the roles of these phosphoevents in period determination by tracking bioluminescence signals in real-time.

### FRQ phosphorylation is detected in *frq^84pA^* but not in *frq^110pA^*

**Fig. 2.**
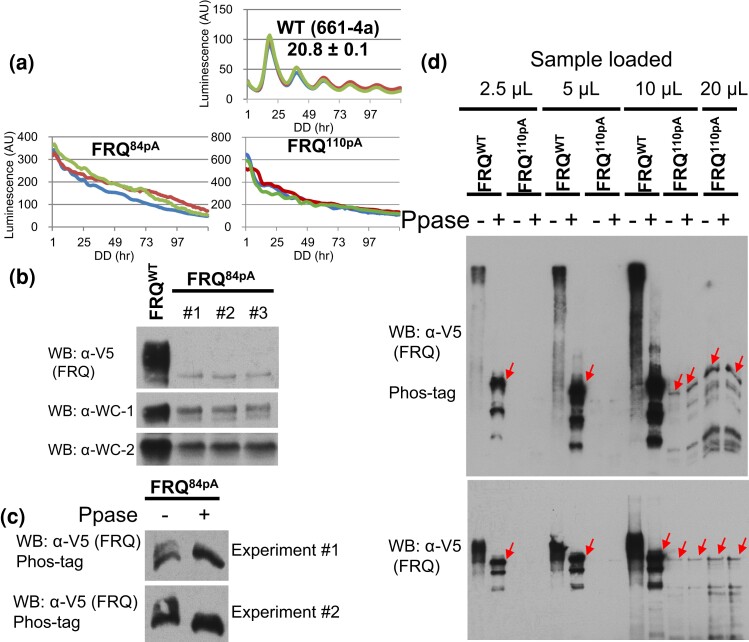
Circadian phenotypes and phosphorylation status of FRQ when all the 84 or 110 phosphosites were eliminated. a) Luciferase assays of *frq^84pA^* and *frq^110pA^* at 25°C in the dark. *frq^84pA^* and *frq^110pA^* bear Ala mutations to all the 84 phosphosites (reported in (Baker *et al.* 2009)) and all the 110 phosphosites from (Baker *et al.* 2009; Tang *et al.* 2009), respectively (Fig. 1a). Strains were synchronized at 25°C in the light, and after transfer to the dark at the same temperature, bioluminescence signals were recorded by a CCD camera every hour. b) FRQ, WC-1, and WC-2 expression in wild-type (WT) and *frq^84pA^* by Western blotting (WB). c) Phos-tag gel analysis of FRQ in WT and *frq^84pA^*. FRQ tagged with V5H6 was immunoprecipitated (abbreviated IP’ed) with V5 resin from a constant light culture at 25°C and then treated with lambda phosphatase (labeled as Ppase) to remove phosphorylation. d) similar to (c), FRQ in *frq^110pA^* was pulled down with V5 resin from a culture grown in constant light at 25°C, lambda phosphatase and its buffer supplied by the vendor were added to the washed resin, and the mixture was incubated at 30°C for removal of phosphorylation. In the gel for Western blot, 2.5, 5, 10, or 20 µl of immunoprecipitated/phosphatase-treated products were loaded per lane; the upper blot was performed with a regular SDS-PAGE gel, while the lower one was done using a Phos-tag gel. Red arrows point to bands of the full-length FRQ after dephosphorylation, and bands below them are S-FRQ and degradation products of FRQ, which should lack part of the *N*-terminus because FRQ detected here by WB against V5 is tagged with V5H6 at its C-terminus.

Although over 100 phosphosites have been reported on FRQ, it is unknown whether they represent the entirety of the phosphoevents on the protein. To this end, we first engineered two *frq* mutants, *frq^84pA^* and *frq^110pA^* in which the 84 phosphosites (Baker *et al.* 2009) and all the 110 phosphosites (Baker *et al.* 2009; Tang *et al.* 2009), respectively, were mutated to Ala. The circadian clock was assayed in a strain bearing a codon-optimized firefly *luciferase* gene driven by the *frq-C-box* at the *his-3* locus (Larrondo *et al*., 2015) in which the endogenous wild-type (WT) *frq* gene was replaced by the engineered *frq* mutants. Compared with WT, both *frq^84pA^* and *frq^110pA^* become arrhythmic with a high amplitude of the luciferase signal (Fig. 2a), suggesting an impaired feedback loop lacking repression of *frq* expression caused by these mutations. The level of FRQ in *frq^84pA^* became extremely low but was detectable compared to that in WT (Fig. 2b). FRQ phosphorylation in *frq^84pA^* was analyzed using a modified Phos-tag system by which single phosphorylation events on WC-1 and WC-2 could be unambiguously resolved (Wang *et al.* 2019). To our surprise, despite elimination of all the 84 phosphorylation sites, robust FRQ phosphorylation in *frq^84pA^* was still detected reproducibly by the Phos-tag assay especially when compared to a lambda phosphatase-treated sample (Fig. 2c), meaning that the 84 phosphosites do not include all major phosphoevents on FRQ. Similar to *frq^84pA^*, the level of FRQ in *frq^110pA^* is dramatically reduced but its phosphorylation totally disappeared, reflected by the same migration pattern of FRQ bands from samples treated with or without phosphatase (Fig. 2d); these data suggest that all major phosphoevents on FRQ that occur under these growth conditions have been directly or indirectly eliminated by the 110 mutations introduced. It is worth noting that FRQ stability is known to increase in mutants disrupting phosphorylation in the *N*-terminal and middle parts of the protein (Baker *et al.* 2009; Tang *et al.* 2009), so the extremely reduced FRQ abundance in *frq^84pA^* and *frq^110pA^* suggests an undesirable side effect caused by the large quantity of mutations that have been introduced, rather than through the elimination of phosphorylation per se.

### *Frq* mutants identify phosphoresidues affecting period lengths

**Fig. 3.**
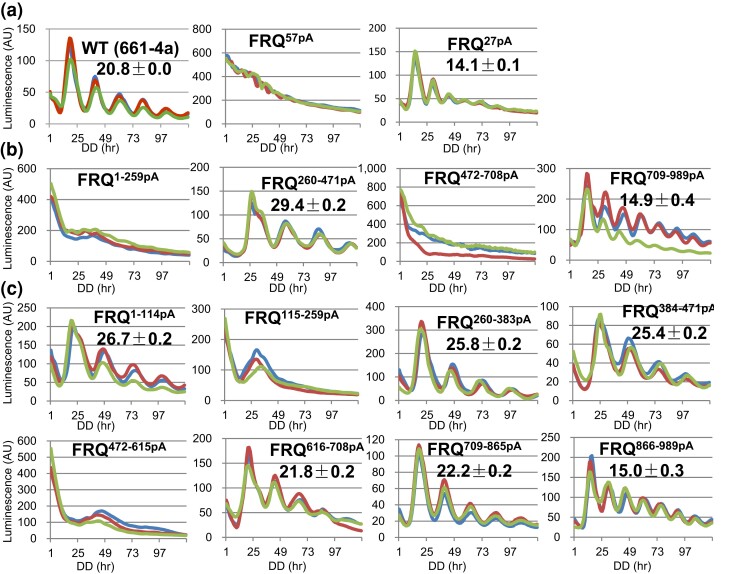
Luciferase analyses of *frq* phosphomutants a) *frq^57pA^* and *frq^27pA^* were analyzed by a luciferase assay at 25°C in the dark. All the 84 phosphorylation sites on FRQ (Baker *et al.* 2009) were dissected into two *frq* mutants: *frq^57pA^* bearing 57 phosphosites in aa 1–682 mutated to Ala altogether and *frq^27pA^* bearing Ala mutations to the remaining 27 phosphosites. Raw data from three replicates (lines in different colors) were displayed, and time (in hours) and arbitrary units of the signal intensity are on the x-axis and y-axis, respectively. In this and subsequent figures, period length was calculated from three or more biological replicates and is reported as the average ± the standard error of the mean (SEM). b) Luciferase analyses of *frq^1–259pA^*, *frq^260–471pA^*, *frq^472–708pA^*, and *frq^709–989pA^* in the dark at 25°C. c) Luciferase analyses of *frq^1–114pA^*, *frq^115–259pA^*, *frq^260–383pA^*, *frq^384–471pA^*, *frq^472–615pA^*, *frq^616–708pA^*, *frq^709–865pA^*, and *frq^866–989pA^* in the dark at 25°C. Strains were cultured in the race tube medium bearing luciferin at 25°C in the light overnight and transferred to darkness at the same temperature for light production recording by a CCD camera.

To directly examine the overall effect of FRQ phosphorylation on period length, we first made two mutants, *frq^57pA^* and *frq^27pA^*, together encompassing all the 84 phosphoresidues (Baker *et al.* 2009) mutated to Ala–*frq^57pA^* encompasses 57 phosphosites falling in amino acids (aas) 1 to 682 of FRQ were mutated to Ala altogether, and *frq^27pA^* bears Ala mutations to the 27 phosphosites in aa 683–989 of FRQ. Consistent with the arrhythmicity observed in *frq^84pA^* and *frq^110pA^* (Fig. 2a), *frq^57pA^* does not develop an oscillating clock while *frq^27pA^* displays a robust rhythm with an extremely decreased period, 14.1 hours (Fig. 3a), shorter than any other *frq* mutants bearing point mutations or deletions to the same region of FRQ, such as *frq^S900A^* (19.5 hours) (Baker *et al.* 2009), *frq^Δ899–989^* (18.7 hours) (Baker *et al.* 2009), *frq* mutants (M14 [21.1 hours], M17 [20.9 hours], M18 [19.9 hours], and M19 [21.0 hours]) (Tang *et al.* 2009), or *frq^C23A^* (18.97 hours) (Cha *et al.* 2011); this suggests an additive effect contributed cooperatively by multiple phosphoevents at the C-terminus of FRQ in controlling the period length. To more specifically elucidate roles of phosphorylations in smaller regions of FRQ, four additional *frq* mutants derived from *frq^110pA^* were generated, each of them containing Ala mutations to phosphosites spanning ∼200–300 amino acids (Fig. 1b). In *frq^1–259pA^*, all phosphorylatable residues between aa 1 and 259 of FRQ were changed to Ala, while keeping the remaining aa 260–989 WT and therefore potentially phosphorylatable; in *frq^260–471pA^*, phosphosites between 260 and 471 were changed to Ala; in *frq^472–708pA^* phosphosites between aa 472 and 708 were changed to Ala; and in *frq^709–989pA^*, phosphosites between aa 709 and 989 were changed to Ala. Luciferase analysis showed that *frq^1–259pA^* and *frq^472–708pA^* exhibit a loss of rhythmicity; *frq^260–471pA^* has an increased period length (29.4 hours), while *frq^708–989pA^* displays a decreased period length (14.9 hours) (Fig. 3b), consistent with the circadian phenotype of *frq^27pA^* (Fig. 3a). *frq^1–259pA^* bears mutations in and near to the coiled-coil domain that is required for FRQ to interact with itself and other core clock components (Cheng *et al.* 2001) as well as mutations near but not within the nuclear localization signal (NLS) (Luo *et al.* 1998), which would seem to explain the lost rhythmicity seen in the mutant. However, that is not the case (see below: *frq^115–193pA^* and *frq^194–220pA^*). Phosphorylation surrounding the coiled coil (CC) and NLS was eliminated in *frq^115–193pA^* and *frq^194–220pA^*, respectively, which showed periods of 20.7 and 26 hours, respectively (Fig. 6a and 6b), suggesting that abolishing phosphorylation within or near to these domains does not completely eliminate FRQ function, and arrhythmicity in *frq^1–259pA^* is not entirely the result of eliminating phosphorylation within and close to CC and NLS. Because L-FRQ alone is sufficient for maintaining a clock at 25°C (Liu *et al.* 1997), the arrhythmicity of *frq^1–259pA^* should not result from disruption of S-FRQ expression, which is also supported by the robust rhythmicity noted in *frq^1–114pA^*, albeit with a longer period (see below).

To separately follow the impact of these phosphoevents, the 110 phosphorylation sites on FRQ were further divided into eight additional *frq* segments (Fig. 1b), which were mutated and analyzed by real-time luciferase assays as above. Consistent with the phenotypes of *frq^1–259pA^* and *frq^472–708pA^*, *frq^115–259pA^* and *frq^472–615pA^* are arrhythmic. *frq^1–114pA^*, *frq^260–383pA^*, and *frq^384–471pA^* show increased period lengths compared to WT with period lengths of 26.7, 25.8, and 25.4 hours, respectively. *frq^616–708pA^* and *frq^709–865pA^* showed ∼WT period lengths. The period length of *frq^866–989pA^* is 15 hours (Fig. 3c), mostly recapitulating the short period observed in *frq^27pA^* (Fig. 3a) and *frq^709–989pA^* (Fig. 3b) and indicating that phosphorylation of the C-terminal tail of FRQ contributes tremendously to period length determination. Expression of FRQ, FRH, WC-1, and WC-2 in all these eight *frq* mutants (Fig. 3c) is comparable to that in WT (Supplementary Fig. 1). Except for *frq^616–708pA^*, the other seven mutants have normal FRQ–FRH interaction (Supplementary Fig. 1). Interaction between FRQ/FRH and WC-1/WC-2 is decreased in *frq^384–471pA^*, *frq^472–615pA^*, and *frq^616–708pA^*, and it becomes undetectable in *frq^115–259pA^* (Supplementary Fig. 1), consistent with the lost rhythmicity seen in the strain. These data indicate that ablation of certain phosphorylations in the *N*-terminal and middle regions of FRQ causes period-lengthening effects; conversely, removal of phosphorylations within the FRQ C-terminus results in an extremely shortened period, suggesting an autoinhibitory role for this C-terminal domain. In agreement with the period changes of the *frq* phosphomutants in Fig. 3c, canonical *frq* alleles except for *frq^1^* at the *N*-terminus of FRQ display a lengthened period, while *frq^2^* (bearing the same mutation as *frq^4^* and *frq^6^* at Ala 895) shows a decreased period (Feldman 1982; Aronson *et al.* 1994a), suggesting that these mutations may impact phosphorylation of other residues, leading to period changes, although they are not phosphorylatable per se or conversely, neighboring phosphorylation events might modulate period lengths via impacting these nonphosphorylatable but functionally crucial residues.

### *frq^866–989pA^* shows a strongly overcompensated clock across a temperature range

**Fig. 4.**
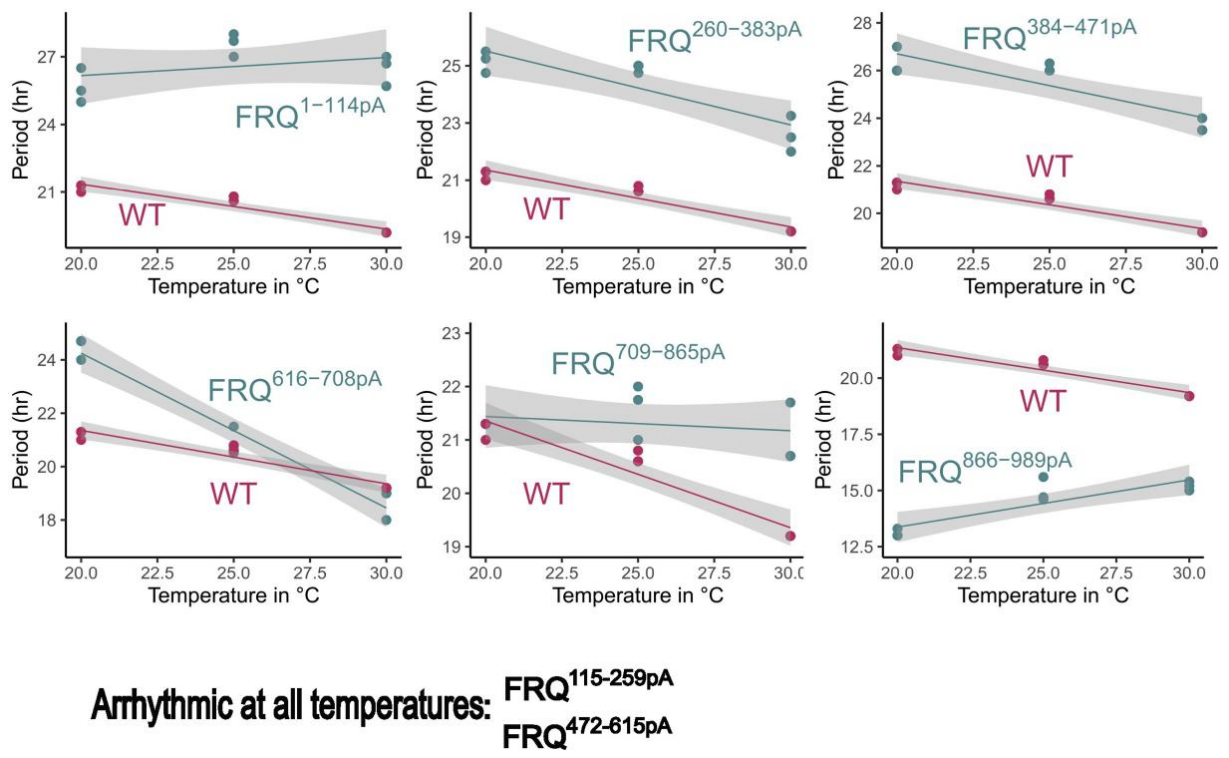
Luciferase analyses of *frq* phosphomutants of *frq^1–114pA^*, *frq^115–259pA^*, *frq^260–383pA^*, *frq^384–471pA^*, *frq^472–615pA^*, *frq^616–708pA^*, *frq^709–865pA^*, and *frq^866–989pA^* at three physiological temperatures, 20, 25, or 30°C. Strains were grown at three temperatures 20, 25, or 30°C in the presence of light and then transferred to the dark for bioluminescence signal recording at the same temperature. Note: the period length of *frq^616–708pA^* at 30°C was calculated using the first two cycles only. Temperature in degrees is on the x-axis, and period length in hours is on the y-axis. Raw data are shown in Supplementary Fig. 2. Statistical significance for the impact of genotype and temperature on period length was determined by a two-way ANOVA analysis for rhythmic strains: *frq^1–114pA^* (*P* = 0.000486), *frq^260–383pA^* (*P* = 0.093818), *frq^384–471pA^* (*P* = 0.034883), *frq^616–708pA^* (*P* = 2.75E-06), *frq^709–865pA^* (*P* = 0.002353), and *frq^866–989pA^* (*P* = 5.38E-08).

The kinases involved in phosphorylation of FRQ, especially CK1 and CK2, have been implicated in controlling temperature compensation of the core oscillator (Liu *et al.* 1997; Mehra *et al.* 2009; Hu *et al.* 2021) in which the circadian period length is only slightly altered across a range of physiological temperatures. Compensation is a conserved characteristic observed across diverse circadian systems. To explore whether the phosphorylation clusters on FRQ regulate the core clock at other temperatures, the eight *frq* phosphomutants in Fig. 3c were further examined at 20, 25, and 30°C: *frq^260–383pA^* and *frq^384–471pA^* show a period trend similar to that seen in WT; *frq^1–114pA^* and *frq^709–865pA^* display constant period lengths across temperatures even more so than WT; *frq^115–259pA^* and *frq^472–615pA^* remain arrhythmic, and *frq^616–708pA^* showed a decreased period at higher temperatures, indicating this strain has an undercompensated clock (Fig. 4 and Supplementary Fig. 2). Interestingly, *frq^866–989pA^* bearing Ala mutations at amino acids 900, 904, 915, 917, 923, 929, 931, 950, 956, 967, and 968 of FRQ demonstrates enhanced period lengths at higher temperatures and therefore has an overcompensated clock (Fig. 4, bottom left), indicating that phosphorylation of the C-terminal tail of FRQ is involved in maintaining period lengths at enhanced temperatures. This result is consistent with a recent publication showing that mutation of three CK2 in vitro-phosphorylated sites not covered in this study, S980, S981, and S982, also result in an increased period at an elevated temperature (Hu *et al.* 2021). Alternatively, these 11 sites are located close to the PEST-2 domain of FRQ (Gorl *et al.* 2001), so their phosphorylation may indirectly impact its function leading to the period adjustment. It is worth noting that the number of mutations introduced to FRQ does not always correlate with the severity of the period alteration. For example, *frq^866–989pA^* bearing 11 mutations displays a dramatically shortened period at 25°C and an overcompensated clock across the three temperatures (Fig. 3c and 4), while *frq^616–708pA^* with 12 mutations still exhibits a WT period at 25°C and an undercompensated oscillator at higher temperatures, while *frq^709–865pA^* carrying nine mutations maintains a WT period at 20, 25, and 30°C (Fig. 3c and 4). *frq^866–989pA^* shows a much stronger period phenotype at the higher temperature than the *frq^Q2^* mutant which bears Ala mutations to four phosphosites 685, 800, 915, and 929 but retains normal temperature compensation (Mehra *et al.* 2009), suggesting that FRQ C-terminal phosphorylations contribute collaboratively to maintaining the period length across temperatures.

### Combination of few key phosphosites on FRQ is required for temperature compensation of the clock

**Fig. 5.**
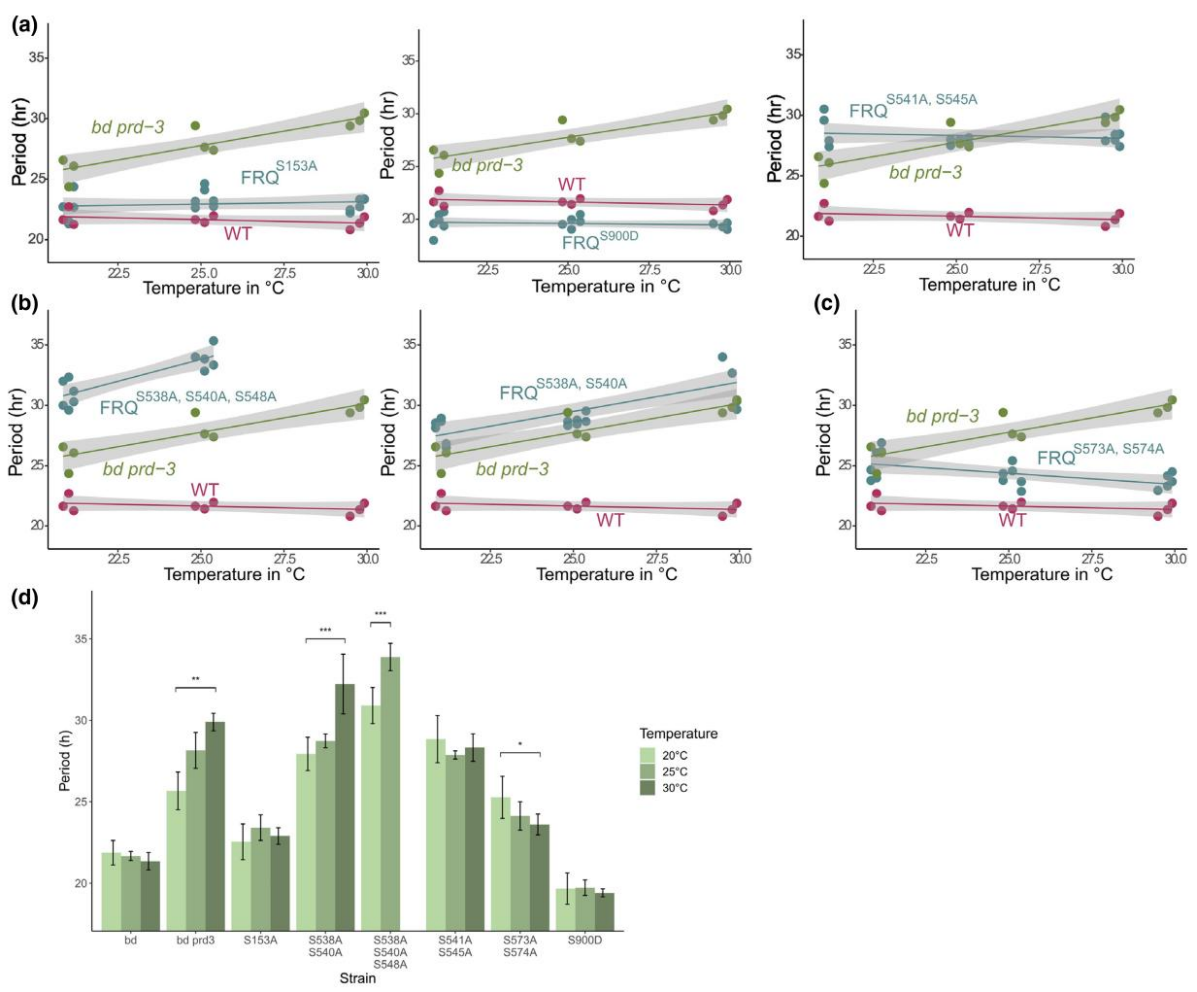
Combination of few key phosphosites on FRQ is required for Normal temperature compensation. FRQ phosphosite mutants from Baker *et al*. (2009) were screened for temperature compensation defects by crossing a transcriptional *frq luciferase* reporter into each strain. Strains were entrained on a 12/12 light dark cycle for 2 days at 25°C, and then transferred to the dark at 20, 25, or 30°C to record luciferase oscillations. The negative control (labeled as WT) was *ras-1^bd^* and the positive control for temperature compensation defects was the classic overcompensation mutant *ras-1^bd^*, *prd-3*. a) Nearly all strains screened showed normal temperature compensation profiles, regardless of their period lengths relative to WT at each temperature. Representative examples show WT, long, and short period lengths with normal temperature compensation (see Supplementary Table 1 for all period length data). b) Two strains were overcompensated against temperature, *frq^S538A, S540A^* and *frq^S538A, S540A, S548A^*. *frq^S538A, S540A, S548A^* was arrhythmic at 30°C. c) One strain, *frq^S573A, S574A^*, was slightly undercompensated against temperature. d) Period lengths of strains depicted in a, b, and c at each temperature tested. Two siblings from each cross were screened, *n* = 3 at each temperature. Student's t-test was used to determine statistical significance between period length at 20°C vs 30°C (25°C vs 30°C for *frq^S538A, S540A, S548A^*). *P*-value of * is ≤ 0.05, ** is ≤ 0.01, and *** is ≤ 0.001. Strains without an asterisk above indicate that the difference is not significant. Supplementary Table 1 lists period lengths for all strains tested, including those not depicted here. Supplementary Fig. 3 shows luciferase traces for strains shown in a, b, and c. Two-way ANOVA was run to test the interaction between genotype and temperature on period length for the following strains: *frq^S153A^* (*P* = 0.459745), *frq^S538A, S540A^* (*P* = 0.00057), *frq^S538A, S540A, S548A^* (*P* = 0.001759), *frq^S541A, S545A^* (*P* = 0.54789), *frq^S573A, S574A^* (*P* = 0.390609), and *frq^S900D^* (*P* = 0.891871).

Given that our mutational analysis of FRQ phosphosites revealed specific domains involved in temperature compensation, we investigated at a more detailed level the involvement of single, double, or triple phosphosites on FRQ in temperature compensation. A subset of the FRQ phosphosite mutants constructed in Baker *et al.* (2009) were crossed to the *C-box-luciferase* reporter targeted to the *csr-1* locus, and two siblings from each cross were screened at 20, 25, and 30°C (*n* = 3 at each temperature) (Supplementary Table 1). The negative control, *ras-1^bd^* (clock WT), had normal temperature compensation, and the positive control, *ras-1^bd^*, *prd-3* (Mehra *et al.* 2009) was overcompensated as expected. Most FRQ phosphosites, when mutated, did not perturb temperature compensation, even when period length was changed (Fig. 5a shows representative examples; Supplementary Table 1 contains period length data at all temperatures for all of the strains tested). However, mutation of S538A & S540A or of S538A & S540A & S548A on FRQ resulted in extreme overcompensation in which period length increased as temperature increased (Fig. 5b). Compared to S538A & S540A, the additional mutation of S548 to Ala increased the period length dramatically and also caused arrhythmicity at 30°C, suggesting that this site acts synergistically with the others in this cluster. Mutation of S573A & S574A caused modest undercompensation (Fig. 5c). Statistical differences between period lengths at low vs high temperatures determined using Student's t-test (Fig. 5d) indicate that of these mutants that were examined, no single phosphosite alone is responsible for period modulation with temperature. Rather, only mutation of a combination of several key phosphosites perturbs temperature compensation, and it appears that undercompensation or overcompensation phenotypes are determined by distinct phosphosites on FRQ.

### Further defining phosphosites in the arrhythmic mutants of *frq*

**Fig. 6.**
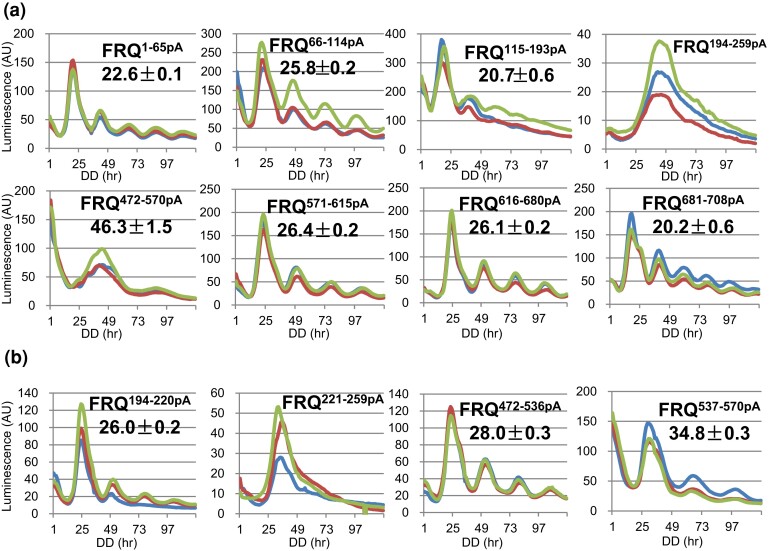
Further dissecting FRQ phosphorylation events falling in amino acids 1–259 and 472–708. a) Luciferase analyses of *frq* phosphomutants, *frq^1–65pA^*, *frq^66–114pA^*, *frq^115–193pA^*, *frq^194–259pA^*, *frq^472–570pA^*, *frq^571–615pA^*, *frq^616–680pA^*, and *frq^681–708pA^* at 25°C. Note: the period length of *frq^472–570pA^* was calculated only from two available circadian cycles. b) Luciferase analyses of *frq^194–220pA^*, *frq^221–259pA^*, *frq^472–536pA^*, and *frq^537–570pA^* at 25°C.

Because eliminating phosphorylation in aa 115–259 or 472–615 resulted in arrhythmicity (Fig. 3c), additional *frq* mutant strains bearing fewer, more select mutations were generated to these and their neighboring regions (Fig. 6a) in order to understand the roles of these phosphoevents in period manipulation. *frq^1–65pA^* carrying nine mutations displayed a WT period length, while *frq^66–114pA^* with eight point mutations showed a long period length similar to that in *frq^1–114pA^*, suggesting that the effect of phosphorylations in aa 1–114 on period length is mainly caused by those in aa 66–114 (Fig. 6a). The period of *frq^115–193pA^* was only slightly shorter than WT, while *frq^194–259pA^* remained arrhythmic, similar to *frq^115–259pA^* (Fig. 6a), indicating the arrhythmicity in *frq^115–259pA^* is due mainly to the loss phosphosites in aa 194–259. It seems that phosphorylation may not impact FRQ dimerization, because the period length of *frq^115–193pA^* remains ∼WT although it bears mutations close to and within the CC domain (aa 143–176) (Cheng *et al.* 2001). Although *frq^472–615pA^* is arrhythmic (Fig. 3c), *frq^472–570pA^* shows a long period of 46.3 hours, which, to our knowledge, is the longest period seen in *frq* phosphomutants to date, and *frq^571–615pA^* shows 26.4 hours (Fig. 6a). *frq^616–680pA^* displays a long period, 26.1 hours, and *frq^681–708pA^* is only slightly shorter (Fig. 6a). *frq^616–708pA^* shows an intermediate period between *frq^616–680pA^* and *frq^681–708pA^*, which suggests an averaging effect of two neighboring phosphorylation clusters on period length. Bearing mutations near the FFC domain, *frq^616–708pA^* has less FRH and WCC complexed with FRQ (Supplementary Fig. 1) but it still maintains a ∼WT period (Fig. 3c), consistent with the evidence that the amount of FRH (Hurley *et al.* 2013) or WCC (Liu *et al.* 2019) in the FFC-WCC is not a determinant of the period length, even though the feedback loop relies on their presence in the complex.

To elucidate why loss of phosphorylation between aa 194 and 259 causes arrhythmicity (Fig. 6a), two additional mutants, *frq^194–220pA^* and *frq^221–259pA^*, were generated and assayed by luciferase analyses. *frq^194–220pA^* has mutations to phosphosites near the NLS (aa 194–199) but is robustly rhythmic, albeit with a longer period length (Fig. 6b), suggesting that phosphorylation does not control the nuclear localization of FRQ required for a functional clock (Luo *et al.* 1998). This is consistent with a prior report that FRQ phosphorylation does not significantly impact its subcellular localization (Cha *et al.* 2011). The arrhythmicity seen in *frq^221–259pA^* (Fig. 6b) may be caused by elimination of sites near FCD1 (Fig. 1a), a domain required for CK1 interaction and phosphorylation of the *N*-terminus of FRQ (Querfurth *et al.* 2011). *frq^472–536pA^* and *frq^537–570pA^* are 6 and 13 hours longer than WT, respectively, but *frq^472–570pA^* is ∼24 hours longer (Fig. 6b), which is significantly longer than the additive lengthening of 19 hours (6 + 13 hours), suggesting that the cumulative effect of phosphorylations on period length can be stronger than the additive effect from constituent parts. *frq^472–536pA^* contains three mutations in and close to one of the only two regions of FRQ predicted to have secondary structures (Fig. 1b). This is also a region that comprises the FCD2, so the lengthened periods of the two mutants (*frq^472–536pA^* and *frq^537–570pA^*) may be due to reduced CK1 interaction, consistent with an observation that the period length is determined by FRQ-CK1 interaction (Liu *et al.* 2019).

### Epistasis analyses of distinct phosphogroups on FRQ

**Fig. 7.**
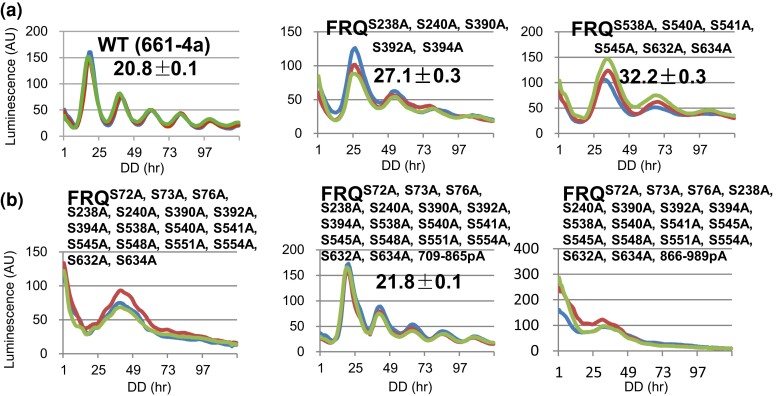
Interplay between FRQ phosphorylations in period determination. a) Luciferase analyses of *frq^S238A, S240A, S390A, S392, S394A^* and *frq^S538A, S540A, S541A, S545A, S632A, S634A^* at 25°C. b) Luciferase analyses of *frq^S72A, S73A, S76A, S238A, S240A, S390A, S392A, S394A, S538A, S540A, S541A, S545A, S548A, S551A, S554A, S632A, S634A^*, *frq^S72A, S73A, S76A, S238A, S240A, S390A, S392A, S394A, S538A, S540A, S541A, S545A, S548A, S551A, S554A, S632A, S634A, 708–865pA^*, and *frq^S72A, S73A, S76A, S238A, S240A, S390A, S392A, S394A, S538A, S540A, S541A, S545A, S545A, S548A, S551A, S554A, S632A, S634A, 865–989pA^* at 25°C.

An intermediate period length has been observed when different FRQ mutations (at nonphosphorylatable residues) were combined intramolecularly; examples include *frq^3^* and *frq^7^* (Aronson *et al.* 1994a), *frq^S548A, S900A^* (Baker *et al.* 2009), and *frq^M9 + 18^* (Tang *et al.* 2009). To check whether this is true for mutants at phosphoresidues in different regions, some of previously reported FRQ phosphomutations were combined together. For instance, both *frq^S238A, S240A^* and *frq^S238A, S240A, S390A, S392, S394A^* each display a period length ∼2 hours longer than WT (Baker *et al.* 2009). The combination of S238A, S240A, S390A, S392A, and S394A would be predicted to be ∼26 hours, close to what we observed experimentally in *frq^S238A, S240A, S390A, S392A, S394A^* (27.1 hours, Fig. 7a). Similarly, mutations in *frq^S538A, S540A^* (5 hours longer than WT [+5 hours]), *frq^S541A, S545A^* (+3 hours), and *frq^S632A, S643A^* (+3 hours) (Baker *et al.* 2009) were all introduced to a single *frq* mutant together, *frq^S538A, S540A, S541A, S545A, S632A, S634A^*, which exhibits a rhythm of 32.2 hours (Fig. 7a), exactly what is anticipated from an additive effect of the three original mutants. Taken together, these data indicate an additive effect of certain FRQ phosphomutations on period length, although this may not be extended to any combinations of FRQ phosphorylations.

To examine the overall effect of individual mutations that alter the period in the same direction, FRQ phosphomutations causing increased period lengths (Baker *et al.* 2009) were together introduced into a single *frq* mutant—*frq^S72A, S73A, S76A, S238A, S240A, S390A, S392A, S394A, S538A, S540A, S541A, S545A, S548A, S551A, S554A, S632A, S634A^*—which, unexpectedly, displays a loss of rhythmicity (Fig. 7b). When this is combined with the mutations in *frq^709–865pA^* (Figs. 3c and 4), the resultant mutant, *frq^S72A, S73A, S76A, S238A, S240A, S390A, S392A, S394A, S538A, S540A, S541A, S545A, S548A, S551A, S554A, S632A, S634A, 709–865pA^*, surprisingly, fully restores rhythmicity to *frq^S72A, S73A, S76A, S238A, S240A, S390A, S392A, S394A, S538A, S540A, S541A, S545A, S548A, S551A, S554A, S632A, S634A^* with a period length almost identical to that in *frq^768–865pA^* (Fig. 7b). However, *frq^S72A, S73A, S76A, S238A, S240A, S390A, S392A, S394A, S538A, S540A, S541A, S545A, S548A, S551A, S554A, S632A, S634A, 866–989pA^* still behaves arrhythmically as *frq^S72A, S73A, S76A, S238A, S240A, S390A, S392A, S394A, S538A, S540A, S541A, S545A, S548A, S551A, S554A, S632A, S634A^* (Fig. 7b). In the second case, *frq^709–989pA^* displays a circadian rhythm of ∼15 hours, which is the same as *frq^866–989pA^* rather than *frq^709–865pA^* (Fig. 3b and 3c), suggesting that the 11 phosphoevents occurring in aa 866–989 are epistatic to the nine found in aa 709–865. Collectively, these data suggest that the interplay between phosphogroups on FRQ can control rhythmicity and period length in diverse ways, including averaging, additive, or epistatic effects.

### Phosphomimetics at S900 could not mimic the effect of phosphorylation at the site

**Fig. 8.**
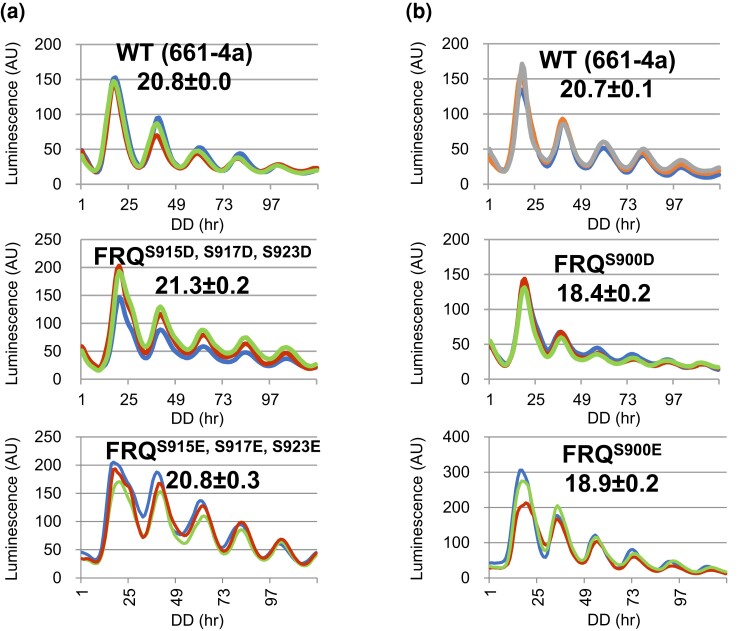
Phosphomimetics of residues on FRQ shows opposite effects. a) *frq^S900D^* and *frq^S900E^* display the same period length as *frq^S900A^* at 25°C. b) *frq^S915D, S917D, S923D^* and *frq^S915E, S9157E, S923E^* show a WT period length at 25°C.

Phosphomimetics by amino acid substitutions like Asp (D) or Glu (E) are a widely used strategy to simulate phosphorylation by constitutively introducing a negative charge into a domain. In *Neurospora*, phosphomimetics have been successfully employed to study phosphorylation of the core clock components, WC-1, WC-2 and FRQ at certain sites, such as *wc-1^S971D^* (Wang *et al.* 2019), *wc-2^15pD^* (Wang *et al.* 2019), and *frq^S548D^* (Baker *et al.* 2009), revealing interesting consequences caused by constant phosphorylation at these sites. To assess whether constitutive phosphorylation at certain sites impacts FRQ activity, several key phosphosites on the protein were mutated to Asp (D) or Glu (E) to mimic the negative charge of the phosphate group. The period length of *frq^S915A, S917A^* and *frq^S923A^* is ∼2 and 1 hour longer than WT, respectively (Baker *et al.* 2009), whereas *frq^S915D, S917D, S923D^* and *frq^S915E, S917E, S923E^* show a WT period (Fig. 8a); similarly, *frq^S548A^* becomes 4-h longer, while *frq^S548D^* maintains a WT period (Baker *et al.* 2009), suggesting that phosphorylation at these sites of FRQ contributes to maintaining the pace of the clock. However, unexpectedly, both *frq^S900D^* and *frq^S900E^* exhibit the same period length (18.4 and 18.9 hours, respectively) (Fig. 8b) as *frq^S900A^* (∼18 hours) (Baker *et al.* 2009), suggesting that the structure of the phosphate group of pS900 plays a more important role than the negative charge that it carries in tuning the FRQ activity. Although the phosphate group and Asp/Glu are both negatively charged, their small structural distinctions may explain the failure of *frq^S900D^* and *frq^S900E^* as phosphorylation mimics and their behavior, instead, like phosphorylation eliminators.

## Discussion

**Fig. 9.**
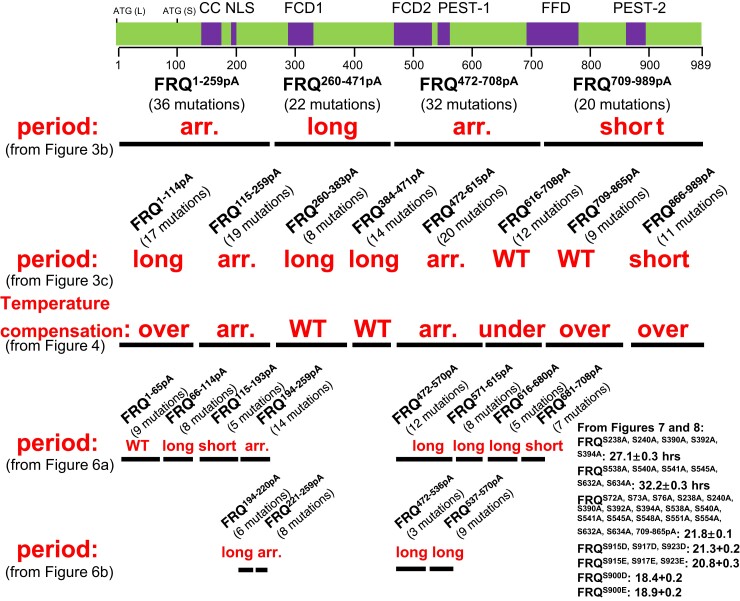
Summary of circadian phenotypes of *frq* phosphorylation mutants generated in this study. The schematic is the same as Fig. 1b with period information at 25°C (in red) and temperature compensation results of the eight indicated strains from 20, 25, and 30°C (in red as well) displayed below the mutant names. The period and temperature compensation results summarized here were derived from Figs. 3b, 3c, 4, 6a, and 6b as indicated in the figure.

FRQ has been predicted to be a largely unstructured protein comprising many disordered regions that make most of its residues exposed and accessible by kinases in the cell (Hurley *et al.* 2013; reviewed in Pelham *et al.* 2020; Marzoll *et al.* 2022b, 2022a), which is consistent with a large number of phosphorylatable residues identified on it. Although over 100 phosphosites on FRQ have been unambiguously documented (Baker *et al.* 2009; Tang *et al.* 2009) and partially confirmed by a recent publication (Horta *et al.* 2019), and Ala mutations to some of these phosphoresidues have been shown to alter period lengths, their functions are still largely unknown due to lack of systematic mutagenesis analyses to all of them. In this study, we generated and studied a large number of *frq* phosphomutants covering all 110 phosphosites, and detailed mutagenetic analyses have allowed circadian roles of these site assigned to different domains of FRQ (summarized in Fig. 9). Excluding those mutations that resulted in arrhythmicity, we found that mutating phosphoresidues in the *N*-terminal or middle regions of FRQ only cause increased or unaltered period lengths while removal of phosphorylated residues at the C-terminus or in the middle (the cluster of S538, S540, and S548 in Fig. 5b) of FRQ results in a decreased or elevated period length, respectively, and an overcompensated circadian clock across a physiological temperature range. Interestingly, either an additive or epistatic effect on rhythmicity has been observed when combining different groups of mutations together.

How is FRQ activity tightly tuned over the course of a day? Recent publications have strongly challenged the model in which the period length is determined by the half-life of FRQ and, instead, support that time-of-day-specific phosphorylation of FRQ finely controls its activity (Baker *et al.* 2009; Larrondo *et al.* 2015; Liu *et al.* 2019; Hu *et al.* 2021). Lacking enzymatic activity, FRQ mainly acts as a molecular platform that recruits kinases to phosphorylate its transcription activator, WCC, thereby closing the feedback loop. An intramolecular interaction between the N- and C-termini of FRQ has been demonstrated (Querfurth *et al.* 2011), which might be weakened or disrupted by progressive phosphorylation at multiple sites over time, leading to decreased interaction or even dissociation between FRQ and its interactors, removal of the repression on WCC, and restarting the next circadian cycle. FRQ phosphorylation can impact its activity through two different ways: Phosphorylation occurring within or close to a domain(s) can directly alter its function and interacting partners. Most phosphosites are located in the disordered regions of FRQ, and modifications at these sites can change the overall structure of FRQ in two ways: (1) by disrupting the intramolecular interaction between its N- and C-termini, which is essential for FRQ activity (Querfurth *et al.* 2011) or (2) by impacting the secondary structure of FRQ and thereby its interactions with its partners (e.g. Baker *et al.*, 2009). If phosphorylation at the *N*-terminal and middle regions of FRQ is not allowed or occurs at a slower pace, then it is plausible that the intramolecular interaction within FRQ will be sustained longer along with the capacity of FRQ in WCC repression, in consonance with the long periods seen in the *frq* mutants (Fig. 3b and 3c). Phosphorylation of the FRQ C-terminal tail plays a role in slowing down the pace of the feedback loop (Fig. 3); if this molecular brake via phosphorylation is broken, FRQ loses its capacity to promote WCC phosphorylation more quickly, causing WCC to regain its transcription activity sooner. This is reflected by the short periods seen in mutants such as *frq^709–989pA^* and *frq^866–989pA^* (Fig. 3b and 3c). High temperatures might be able to compensate for the loss of these phosphorylations, so the shortened period gets rescued to some extent at a higher temperature (Fig. 4).

Both FRQ and its transcriptional activator WCC are subject to extensive phosphorylations in a circadian cycle, and, similarly, activities of both protein complexes are finely controlled by multiple phosphoevents (Baker *et al.* 2009; Tang *et al.* 2009; Wang *et al.* 2019). For example, WCC transcription activity in the dark is completely inhibited only when a small group of sites on both WC-1 and WC-2 are simultaneously phosphorylated (Wang *et al.* 2019), while a large number of phosphoevents on WCC play little or no role in the core clock but only act on lowering expression of *frq* and clock-controlled genes (namely circadian amplitude) (Wang *et al.* 2019). Similarly, although FRQ is also heavily phosphorylated at numerous sites over time, to date no single phosphomutant of *frq* has been found to be constantly active or inactive in a circadian cycle, suggesting that FRQ activity is indeed determined by multiple phosphoevents. However, an obvious difference between phosphorylation on FRQ and WCC is that most *wcc* phosphomutants do not show substantially altered period lengths (Wang *et al.* 2019), whereas a large quantity of *frq* phosphomutants spanning the whole protein display period changes (Mehra *et al.* 2009; Baker *et al.* 2009; Tang *et al.* 2009; Larrondo *et al.* 2015). These observations agree with a model wherein complexing with FRH, FRQ serves as a platform recruiting kinases to phosphorylate and inhibit WCC, so multiple domains of FRQ participate in interactions with other proteins, including FRH, CKI, and FRQ itself via its FFD, FCD, and CC domains (Fig. 1b), respectively, as well as multiple regions for association with WCC (data not shown). Correspondingly, phosphorylations near or within these regions may directly or indirectly regulate these interactions. FRQ-dependent repression on WCC mainly targets the DNA-binding domain and its nearby regions of WCC (Wang *et al.* 2016, 2019), which explains why mutations to phosphosites in other parts of WCC do not dramatically impact the period length.

FRQ phosphorylation dynamics have been investigated by quantitative mass spectrometric analyses including stable isotope labeling by amino acids in cell culture (SILAC) (Baker *et al.* 2009) and N^15^/N^14^ isotope labeling (Tang *et al.* 2009). A cluster of residues surrounding the PEST-2 region (near aa 795–929) becomes hyperphosphorylated at CT8 when the level of new FRQ and thus its activity begins to increase. Eliminating phosphorylation in 709–989 (*frq^709–989pA^*) results in a short period (Fig. 3b), suggesting that phosphorylation in this region may be required for FRQ to repress WCC. Sites specific to the *N*-terminus of L-FRQ become phosphorylated at CT16, a late time point in a circadian cycle; sites in the PEST-1 domain (aa 537–558) become hyperphosphorylated later, peaking at CT12, suggesting that these phosphorylations may function in inhibiting FRQ activity. Consistent with these, *frq^1–114pA^* and *frq^537–570pA^* develop long periods of 26.7 and 34.8 hours, respectively (Figs. 3c and 6b). Phosphorylation of aa 211–257 peaks earlier and decreases relatively over time, suggesting that the dynamics of phosphorylation at these regions correlates with and may impact the change of FRQ activity in a circadian cycle (Baker *et al.* 2009), supported by the arrhythmicity seen in *frq^221–259pA^* (Fig. 6b). Due to scarcity of purified FRQ for in vitro studies and potential ionization issues of peptides bearing multisite phosphorylations in mass spectrometry, whether phosphorylation of FRQ at many sites changes in concert in a circadian cycle is still largely unknown, which restricts our understanding of the role of time-specific phosphoclusters on FRQ.

Results in this work may inform understanding of mammalian and insect clocks, many facets of which are also built on time-specific multisite phosphorylation events to the key components (reviewed in Brenna and Albrecht 2020). PER/TIM in *Drosophila* and PERs/CRYs in mammals act as the negative elements in the negative feedback loop by inhibiting Clk/Cyc and CLOCK/BMAL1 activities, respectively, terminating their own expression and thereby closing the circadian negative feedback loop. Similar to FRQ and WCC in *Neurospora*, PER/TIM and PERs/CRYs also undergo extensive phosphorylation, and phosphorylation of PER/TIM and PERs/CRYs has been shown to be a critical mechanism in controlling both the fly and mammalian clocks (Chiu *et al.* 2008, 2011; Lamia *et al.* 2009; Top *et al.* 2016; Cao *et al.* 2021; Cai *et al.* 2021; An *et al.* 2022). The strategy adopted here to progressively dissect scores of phosphosites on FRQ might be applicable to facilitating identification of essential phosphoevents on core clock components in other systems.

Lastly, we noted that in the case of FRQ phosphorylation at S900, an aspartic acid or glutamic acid substitution could not faithfully mimic the effect of phosphorylation (Fig. 8b); also, in a few mutants, large numbers of mutations introduced to FRQ might result in undesirable side effect(s) to the protein beyond phosphorylation elimination. These data provide a caveat to the simple interpretation of any phosphosite mutation.

## Supplementary Material

jkac334_Supplementary_Data

## Data Availability

The *Neurospora* strains generated in this study are available upon request. Supporting material is deposited at G3 online. All data used to draw conclusions of the article have been provided within the figures and tables. Supplemental material available at G3 online.

## Notes

*Communicating editor: M. Sachs*
