# Supplementary material for: Functional analysis of 110 phosphorylation sites on the circadian clock protein FRQ identifies clusters determining period length and temperature compensation
Source: G3 (Bethesda). 2022 Dec 20;13(2):jkac334. doi: 10.1093/g3journal/jkac334 (PMC9911066; doi:10.1093/g3journal/jkac334)
Supplement: jkac334_Supplementary_Data [file jkac334_supplementary_data.zip › Supporting_Figure_Legends_G3-2022-403808.docx]

**Supporting Figure 1**

Expression of FRQ, FRH, WC-1, and WC-2 and their interactions in *frq^1-114pA^*, *frq^115-259pA^*, *frq^260-383pA^*, *frq^384-471pA^*, *frq^472-615pA^*, *frq^615-708pA^*, *frq^708-865pA^*, and *frq^865-989pA^* by immunoprecipitation. Strains were cultured at 25 °C in the light, and V5 IP was performed using centrifugation-cleared lysate. Except for WT (untagged), FRQ in all other strains bear a V5H6 tag at their C-termini for detection and immunoprecipitation.

**Supporting Figure 2**

Raw luciferase data of *frq^1-114pA^*, *frq^115-259pA^*, *frq^260-383pA^*, *frq^384-471pA^*, *frq^472-615pA^*, *frq^616-708pA^*, *frq^709-865pA^*, and *frq^866-989pA^* at 20, 25, and 30 °C from three replicates. These strains were synchronized at 20, 25, or 30 °C in the light overnight and in the following day transferred to darkness at the same temperature used in synchronization. Luciferase signals were immediately recorded by a CCD camera after transfer of the strains to the dark.

**Supporting Figure 3**

Raw luciferase data of strains in Figure 5 at 20, 25, and 30 °C from three replicates.

**Supporting Table 1** Raw period data and statistics of luciferase analyses in Figure 5.
