# Supplementary material for: Functional analysis of 110 phosphorylation sites on the circadian clock protein FRQ identifies clusters determining period length and temperature compensation
Source: G3 (Bethesda). 2022 Dec 20;13(2):jkac334. doi: 10.1093/g3journal/jkac334 (PMC9911066; doi:10.1093/g3journal/jkac334)
Supplement: jkac334_Supplementary_Data [file jkac334_supplementary_data.zip › Supporting_Figures_G3-2022-403808.pptx]

## Slide 1
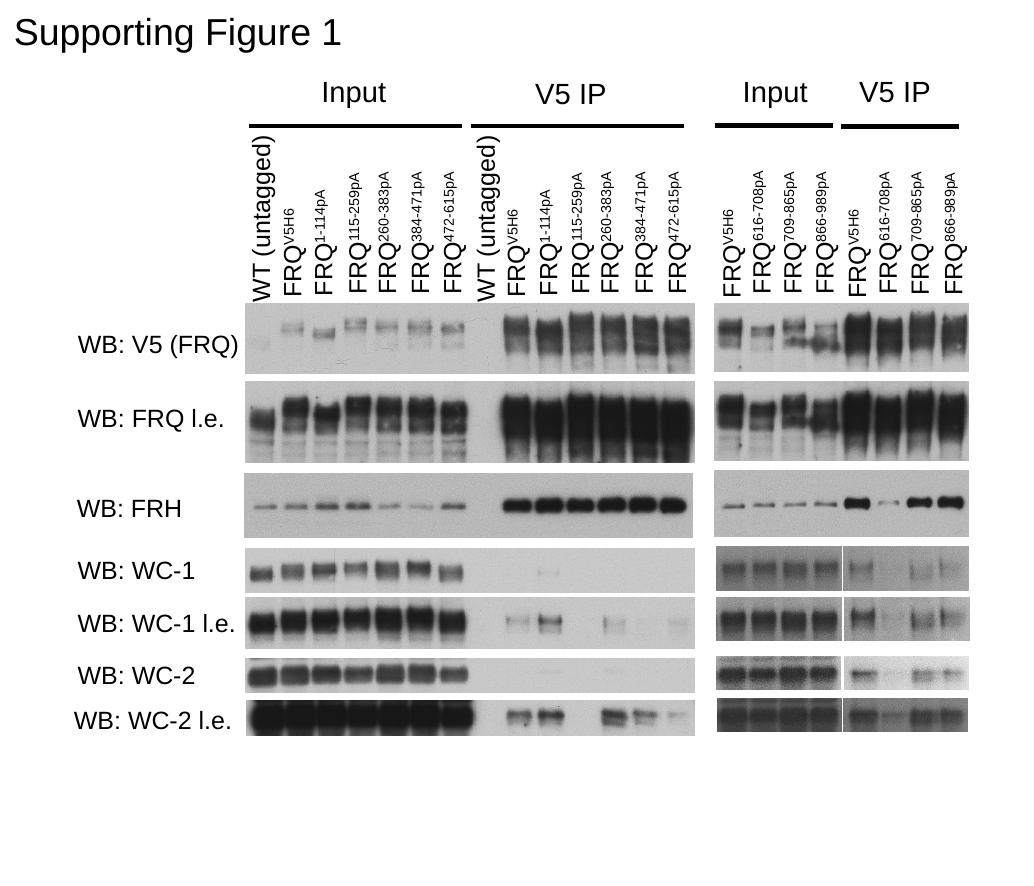

Supporting Figure 1
Input
V5 IP
Input
V5 IP
WT (untagged)
WT (untagged)
FRQ616-708pA
FRQ616-708pA
FRQ709-865pA
FRQ866-989pA
FRQ260-383pA
FRQ384-471pA
FRQ472-615pA
FRQ260-383pA
FRQ384-471pA
FRQ472-615pA
FRQ115-259pA
FRQ115-259pA
FRQ709-865pA
FRQ866-989pA
FRQ1-114pA
FRQ1-114pA
FRQV5H6
FRQV5H6
FRQV5H6
FRQV5H6
WB: V5 (FRQ)
WB: FRQ l.e.
WB: FRH
WB: WC-1
WB: WC-1 l.e.
WB: WC-2
WB: WC-2 l.e.

## Slide 2
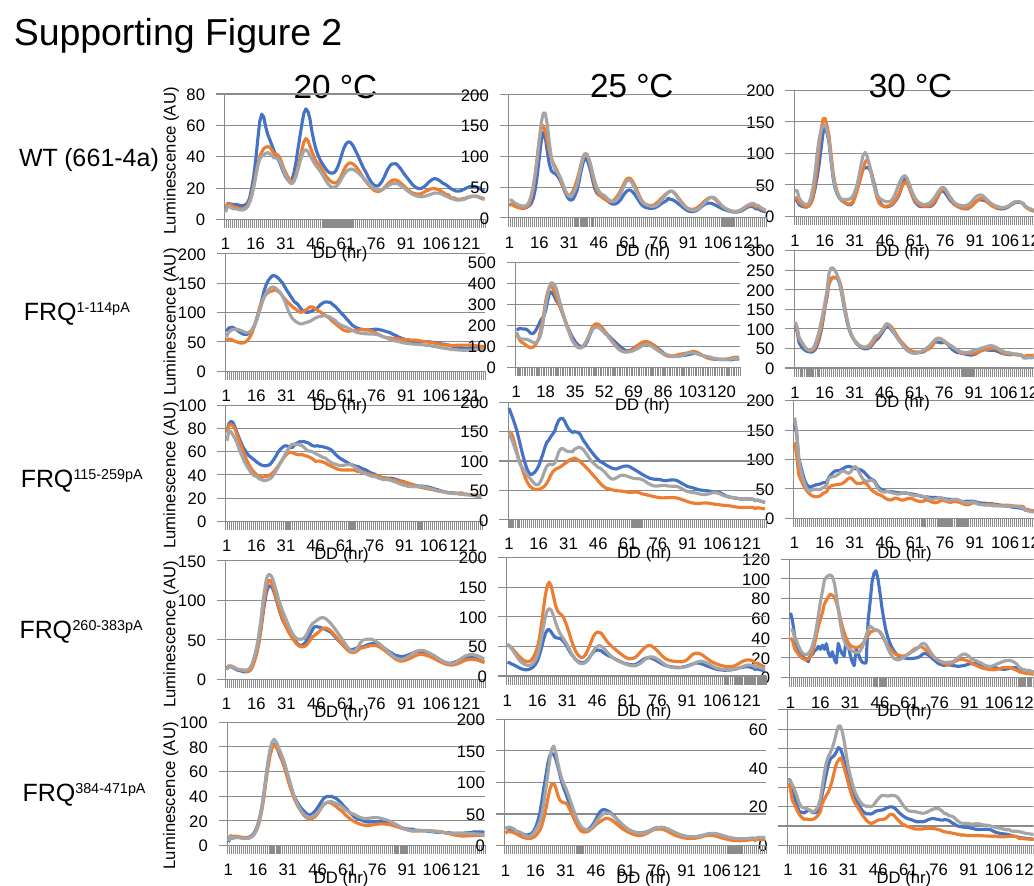

Supporting Figure 2
25 °C
30 °C
# 20 °C
### Chart
| Category | | | |
|---|---|---|---|
### Chart
| Category | | | |
|---|---|---|---|
### Chart
| Category | | | |
|---|---|---|---|WT (661-4a)
Luminescence (AU)
DD (hr)
DD (hr)
DD (hr)
### Chart
| Category | | | |
|---|---|---|---|
### Chart
| Category | | | |
|---|---|---|---|
### Chart
| Category | | | |
|---|---|---|---|FRQ1-114pA
Luminescence (AU)
DD (hr)
DD (hr)
DD (hr)
### Chart
| Category | | | |
|---|---|---|---|
### Chart
| Category | | | |
|---|---|---|---|
### Chart
| Category | | | |
|---|---|---|---|FRQ115-259pA
Luminescence (AU)
DD (hr)
DD (hr)
DD (hr)
### Chart
| Category | | | |
|---|---|---|---|
### Chart
| Category | | | |
|---|---|---|---|
### Chart
| Category | | | |
|---|---|---|---|FRQ260-383pA
Luminescence (AU)
DD (hr)
DD (hr)
DD (hr)
### Chart
| Category | | | |
|---|---|---|---|
### Chart
| Category | | | |
|---|---|---|---|
### Chart
| Category | | | |
|---|---|---|---|FRQ384-471pA
Luminescence (AU)
DD (hr)
DD (hr)
DD (hr)

## Slide 3
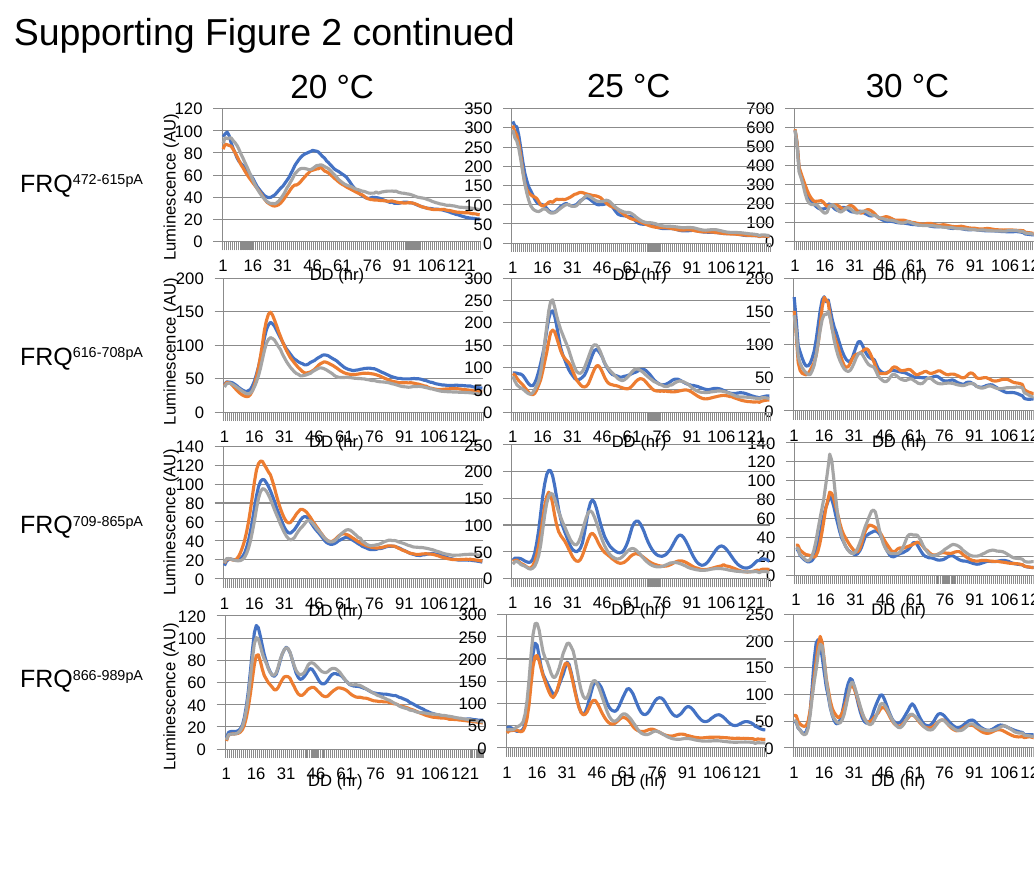

Supporting Figure 2 continued
25 °C
30 °C
# 20 °C
### Chart
| Category | | | |
|---|---|---|---|
### Chart
| Category | | | |
|---|---|---|---|
### Chart
| Category | | | |
|---|---|---|---|FRQ472-615pA
Luminescence (AU)
DD (hr)
DD (hr)
DD (hr)
### Chart
| Category | | | |
|---|---|---|---|
### Chart
| Category | | | |
|---|---|---|---|
### Chart
| Category | | | |
|---|---|---|---|FRQ616-708pA
Luminescence (AU)
DD (hr)
DD (hr)
DD (hr)
### Chart
| Category | | | |
|---|---|---|---|
### Chart
| Category | | | |
|---|---|---|---|
### Chart
| Category | | | |
|---|---|---|---|FRQ709-865pA
Luminescence (AU)
DD (hr)
DD (hr)
DD (hr)
### Chart
| Category | | | |
|---|---|---|---|
### Chart
| Category | | | |
|---|---|---|---|
### Chart
| Category | | | |
|---|---|---|---|FRQ866-989pA
Luminescence (AU)
DD (hr)
DD (hr)
DD (hr)

## Slide 4
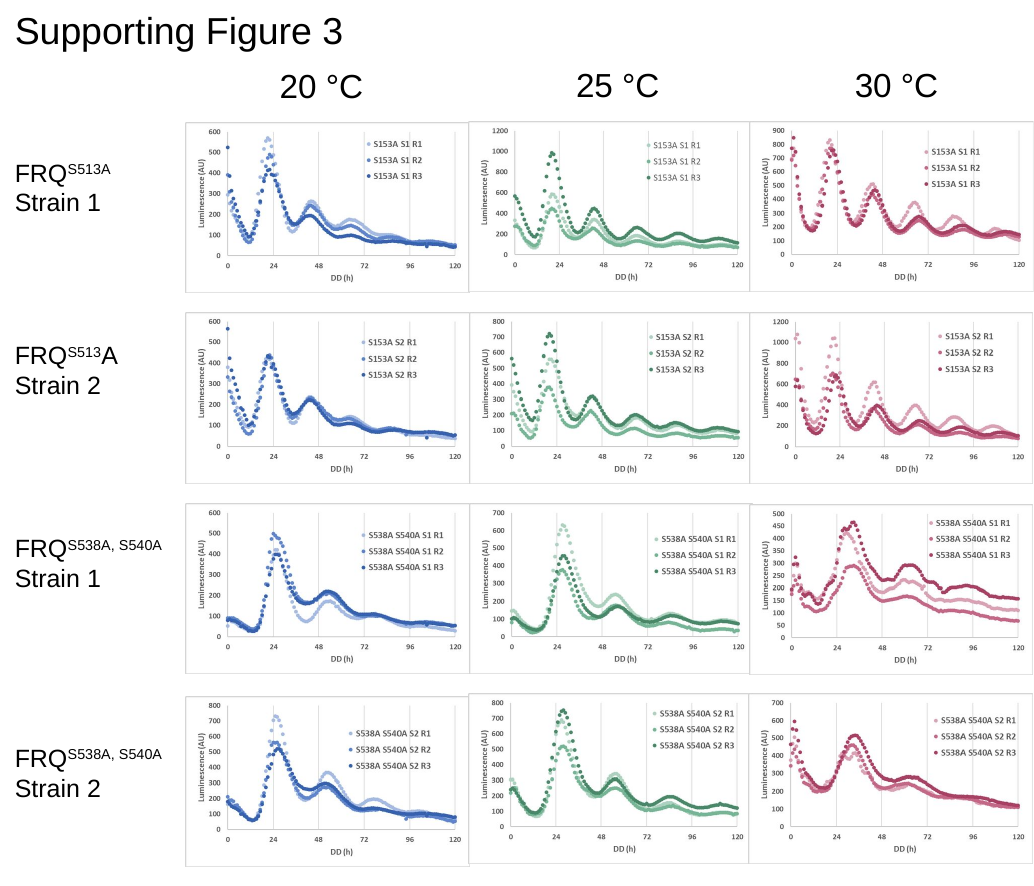

Supporting Figure 3
25 °C
30 °C
# 20 °C
FRQS513A Strain 1
FRQS513A Strain 2
FRQS538A, S540A Strain 1
FRQS538A, S540A Strain 2

## Slide 5
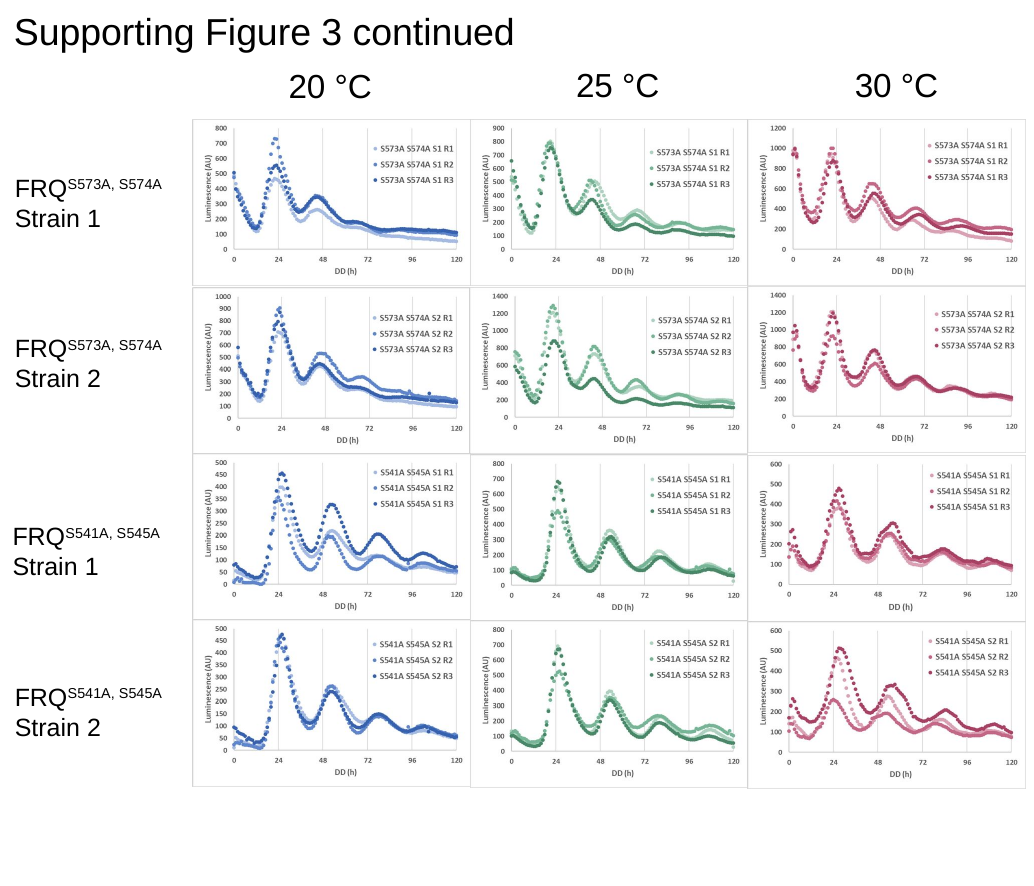

Supporting Figure 3 continued
25 °C
30 °C
# 20 °C
FRQS573A, S574A Strain 1
FRQS573A, S574A Strain 2
FRQS541A, S545A Strain 1
FRQS541A, S545A Strain 2

## Slide 6
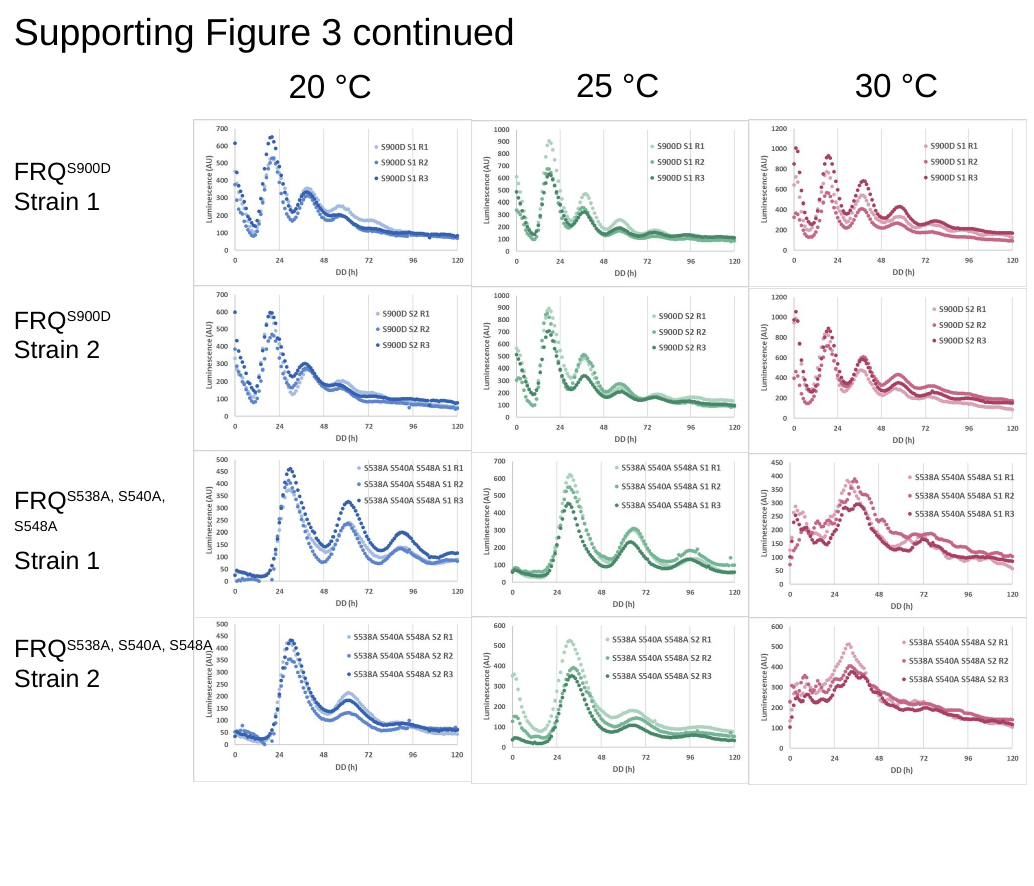

Supporting Figure 3 continued
25 °C
30 °C
# 20 °C
FRQS900D
Strain 1
FRQS900D
Strain 2
FRQS538A, S540A, S548A
Strain 1
FRQS538A, S540A, S548A
Strain 2

## Slide 7
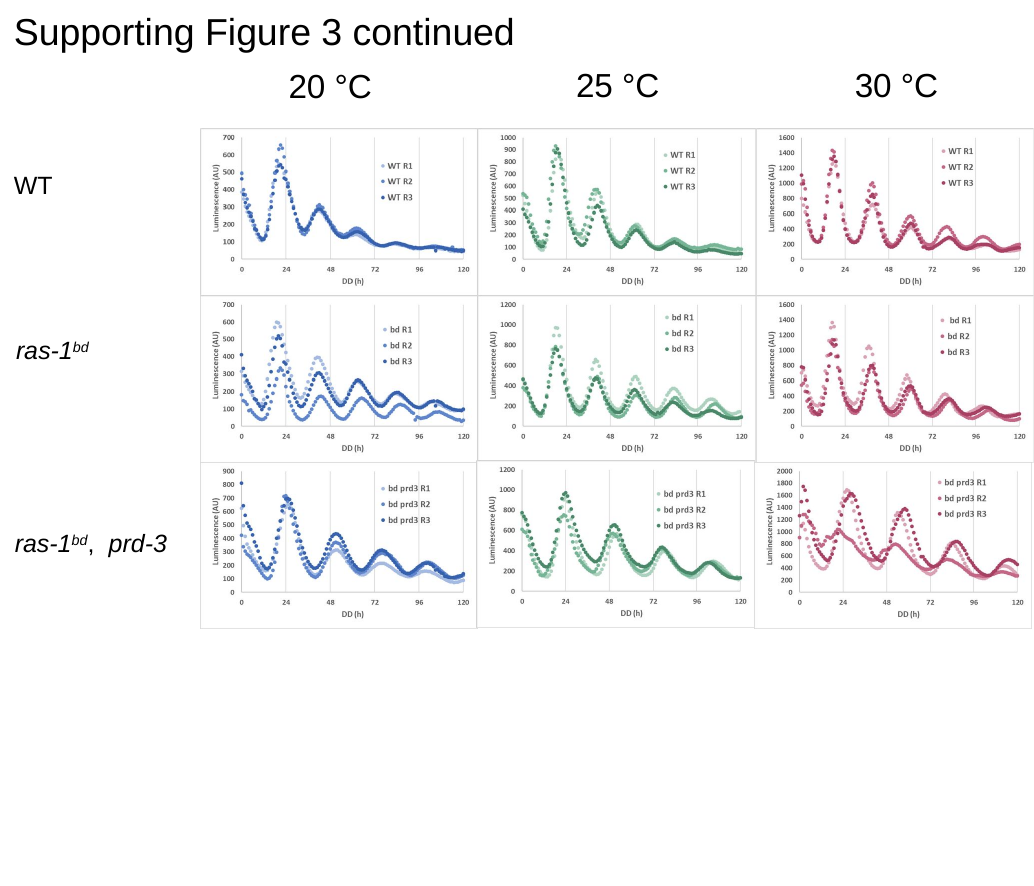

Supporting Figure 3 continued
25 °C
30 °C
# 20 °C
WT
ras-1bd
ras-1bd, prd-3
